# Exclusive enteral nutrition versus corticosteroids for treatment of pediatric Crohn’s disease: a meta-analysis

**DOI:** 10.1007/s12519-018-0204-0

**Published:** 2019-01-21

**Authors:** Yu Yu, Kang-Chen Chen, Jie Chen

**Affiliations:** 1 Department of Gastroenterology, Children’s Hospital, Zhejiang University School of Medicine, 3333 Binsheng Road, Binjiang District, Hangzhou, 310051 China; 2 First Affiliated Hospital, Zhejiang University School of Medicine, 79 Qingchun Road, Shangcheng District, Hangzhou, 310002 China

**Keywords:** Children, Corticosteroids, Crohn’s disease, Exclusive enteral nutrition, Inflammatory bowel disease, Nutrition

## Abstract

**Background:**

Many studies have examined the effects of exclusive enteral nutrition (EEN) in children with Crohn’s disease (CD), but corticosteroids are considered a superior therapy and are frequently used in China. This meta-analysis aims to compare the efficacy of EEN with corticosteroids in treating pediatric CD.

**Methods:**

A comprehensive retrieval from medical databases, including PubMed, EMBASE, MEDLINE, Web of Science, Wanfang data, VIP and CNKI, was performed using the search terms “diet therapy”, “exclusive enteral nutrition”, “Crohn’s disease”, “inflammatory bowel diseases”, “child” and “pediatrics” from January 1990 to April 2017.

**Results:**

We included 18 studies from 1329 identified sources in this meta-analysis. EEN was as effective as corticosteroids in inducing remission rate of children suffering from CD (OR = 1.35; 95% CI 0.90, 2.10; *P *= 0.14). Nevertheless, patients who received EEN were more likely to achieve both endoscopic mucosal healing (OR = 5.24; 95% CI 2.06, 13.37; *P *= 0.0005) and histological mucosal healing (OR = 4.78; 95% CI 1.89, 12.08; *P *= 0.0009) than those who received corticosteroids; the Pediatric Crohn’s Disease Activity Index was lower [mean difference (MD) = − 3.67; 95% CI − 4.91, − 2.43] and weight gain was higher (MD = 1.92; 95% CI 0.02, 3.83; *P *= 0.05) in those patients who received EEN than in those who received corticosteroids. No difference was found in relapse rate (OR = 0.57; 95% CI 0.25, 1.29; *P *= 0.18), height for age or body mass index between the patients treated with EEN and corticosteroids at the 1-year end point.

**Conclusions:**

This meta-analysis reveals that there is no significant difference between EEN and corticosteroids in the efficacy of inducing remission rate of CD in a pediatric population, but EEN is superior to corticosteroids in improving short-term mucosal inflammation and reducing the PCDAI index.

**Electronic supplementary material:**

The online version of this article (10.1007/s12519-018-0204-0) contains supplementary material, which is available to authorized users.

## Introduction

Crohn’s disease (CD) is a chronic relapsing inflammatory condition of the gastrointestinal tract. Causes of CD still remain unclear, but it is thought to result from an interaction of individuals’ genetics factors, epigenetic factors, microbial exposure, immune response and environment factors [1–4]. Approximately, 25% of patients are diagnosed with CD before the age of 18 years [5], and the incidence of pediatric CD is increasing in both developed and developing nations [6, 7]. The goals of treatment in pediatric CD are to induce and maintain remission, relieve symptoms and optimize growth, while minimizing side effects [8].

After an accidental discovery in the 1970s that exclusive enteral nutrition (EEN) could induce remission of CD, EEN has provided an innovative way to induce remission and optimize nutrition following diagnosis [9]. In 2014, the European Society of Pediatric Gastroenterology, Hepatology and Nutrition (ESPGHAN) and the European Crohn’s and Colitis Organization (ECCO) issued revised consensus guidelines, recommending that EEN be considered as the first-line induction therapy for children with CD [10]. For induction of remission, patients were treated with EEN via the oral route or nasogastric tube feeding for approximately 6–8 weeks. Additionally, only chewing gum and water were allowed [11–13]. The dietary sources of protein included elemental, semi-elemental and polymeric diets, but various levels of proteins or lipids seem to not have much impact on efficacy [14–16].

Corticosteroids have been considered a major therapeutic option to induce remission in patients with active CD, achieving a clinical remission in 60–91% of treated patients [1]. However, side effects of corticosteroids, such as Cushing appearance, bone demineralization and severe growth retardation, can be harmful to children’s natural physical development [17]. In addition, evidence-based medicine supports that EEN therapy has fewer adverse events and lower side effect rates than corticosteroids [18]. EEN therapy was also suggested to be more effective in children than adults [19].

Two meta-analyses [18, 20] concluded that EEN, as a primary therapeutic approach for CD, showed no significant difference from corticosteroids in inducing clinical remission. In contrast, two other meta-analyses [21, 22] concluded that patients’ remission rates with corticosteroids were statistically superior to that with EEN. Although many studies have compared short-term remission rates between patients treated with corticosteroids and EEN, few studies have focused on the relapse rate in long-term follow-up, especially in children. Endoscopic sustained mucosal healing in Crohn’s disease was reported to be associated with longer remission time, less inflammatory activity and decreased hospitalization rates [23, 24]; corticosteroids were thought to be associated with poor mucosal healing [25], while EEN was effective in inducing mucosal remission [26]. Few meta-analyses have been performed comparing the differences in efficacy of these two therapies on mucosal healing, and we still do not know whether EEN or corticosteroids will prolong the time that pediatric patients with CD remain in remission.

EEN versus placebo-controlled experiments cannot be carried out in pediatric patients with CD because of difficulty in passing ethical review, and any “placebo” which was nutritionally complete to sustain nutrition during treatment would be regarded as EEN. As a result, most studies have chosen corticosteroid therapy as the control group. In this meta-analysis, we compared the remission rates between two therapy groups at 8 weeks and relapse rates at 1 year. We also included more high-quality studies and further evaluated the effects of two therapeutic strategies on mucosal healing, nutritional status and growth patterns.

## Methods

### Data sources and search strategy

We conducted literature retrieval on PubMed, EMBASE and MEDLINE, Web of Science from January 1990 to April 2017. The search terms “diet therapy” and “exclusive enteral nutrition” were combined using “OR”, the search terms “inflammatory bowel diseases” and “Crohn’s disease” were combined using “OR”, and the search terms “pediatric” and “child” were combined using “OR”. These three groups were then combined using “AND”. For example, searching in PubMed: ((((pediatric[MeSH]) OR child[MeSH])) AND ((exclusive enteral nutrition[MeSH]) OR diet therapy[MeSH])) AND ((Crohn disease[MeSH]) OR inflammatory bowel diseases[MeSH]). Filters: publication date from 1990/01/01 to 2017/04/01. Language restrictions were not imposed. We also searched the literature in Wanfang data, VIP and China National Knowledge Internet with Chinese words for the same keywords and the same time span.

### Selection criteria

Two investigators (YY and CK) independently screened the titles, abstracts and full texts using the search strategy mentioned above. We included studies that: (1) were RCTs or observational; (2) enrolled pediatric patients (under the age of 18 years) with CD; and (3) compared systemic corticosteroid drugs (prednisone, prednisolone) with EEN (polymeric formula, semi-elemental formula or elemental formula). Patients in the EEN arm were not to receive any other medication, and patients receiving corticosteroids were to be treated only with corticosteroids, or the same treatments were to be taken in both comparator arms in similar ways. Studies were excluded in any case when articles (1) did not give a precise definition of remission, (2) provided insufficient data for the outcomes of interest or (3) did not contain at least one clearly defined corticosteroids comparator arm. If a literature result was reported several times, we included the study that corresponded to the longest duration or had the largest sample size.

### Outcome measures

The primary outcome measures were induced remission rate (percentage of subjects achieving remission after 6–8 weeks of treatment) and relapse rate (percentage of subjects relapsing at 1-year end point of follow-up). The secondary outcomes were collected at baseline and after the induced treatment, e.g., inflammation index including the Pediatric Crohn’s Disease Activity Index (PCDAI) [27], C-reactive protein (CRP) and erythrocyte sedimentation rate (ESR); growth parameters such as weight and length; mucosal healing [endoscopic lesions were assessed according to a validated score standard (Crohn’s Disease Endoscopic Index of Severity, CDEIS) [28] and histological lesions were assessed according to a scoring system previously validated [29]. The data on height for age and body mass index (BMI) were collected both at baseline and at the 1-year end point. Endoscopic mucosal healing or histological mucosal healing was separately defined as a decrease in endoscopic or histological scores by 50% or more when compared with baseline values.

### Quality assessment

We abstracted the following data from each study: first author, year of publication, origin, interventions and control groups (drug and dosage), participants’ characteristics (number of each group, age) and underlying condition. We used the Newcastle–Ottawa scale to assess the quality of observational studies. This assessment had three sections (selection, comparability and exposure, respectively) and altogether eight items. Studies with a score less than 5 in the present study were excluded from the final analysis [30]. We also evaluated the bias in randomized controlled trials using the Jadad scale, which included the evaluation of randomization, blinding of outcome assessment and description of withdrawals and dropouts. Studies with a Jadad score more than 3 were regarded as high quality and would be included in the final analysis [31].

We analyzed the results of the RCTs, prospective cohort studies and retrospective cohort studies separately to determine whether the results from the non-RCTs affected our calculation of obvious heterogeneity or produced a different outcome from the more robust RCTs. Because there was a mild heterogeneous effect across strata when we compared each study type, respectively, with our main result, we concluded that it was appropriate to combine study types as hypothesis generating rather than confirmatory analysis.

### Statistical analysis

All the meta-analyses were conducted using Review Manager 5.3. The odds ratio (OR) [32] with 95% confidence intervals (CI) [33] was applied to analyze dichotomous variables, and mean difference (MD) [34] with a 95% CI was used to analyze continuous ones. If continuous data were presented in mean and standard deviation (SD) [35] of base and final, we would use statistical algorithms to calculate the difference value’s mean and SD.

The Chi-square test was used to evaluate the heterogeneity with significance set at the *P *< 0.05 level, and the *I*^2^ statistic value was interpreted as three separate levels: 25% (low heterogeneity), 50% (moderate heterogeneity) and 75% (high heterogeneity) [36]. The random-effect model was conducted when the heterogeneity between studies was too high; otherwise, the fixed-effect model was used [37].

## Results

### Literature research and characteristic of studies

**Fig. 1:**
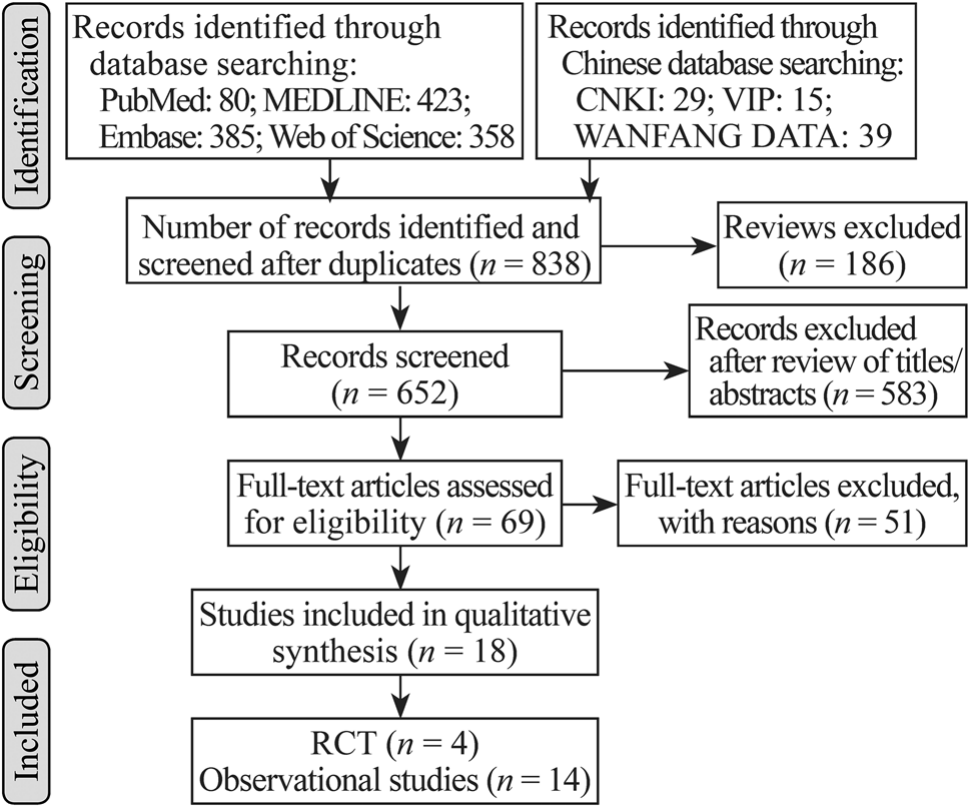
Flow diagram of trials for inclusion in the systematic review. *n* number of records

A total of 1329 publications retrieved from the database were scanned for relevance. After reviewing, we included 69 articles that potentially met inclusion criteria. After these full texts were studied, the bibliographies were checked. Overall, 4 RCTs [38–41] and 14 observational studies [42–55] that reached our inclusion criteria constituted the base of our analysis. Three of the observational studies were abstracts that provided necessary data for inclusion in the meta-analysis (Fig. 1, flow chart). The characteristics of the included studies are shown in Table 1.

**Table 1:** Characteristics of included studies

References	Origin	Study groups, dose, number of patients	Age at diagnosis (y)	Patients	Treatment time (wk)	Clinical remission	Quality score
Randomized controlled trail							
Terrin et al. [38]	Italy	EEN: extensively hydrolyzed formula 50–60 kcal/kg/d (*n *= 10). CS: prednisolone 1.6 mg/kg/d and mesalazine (*n* = 10)	12.4 (7–17)	Children with active CD	8	PCDAI < 10	5
Thomas et al. [39]	England	EEN: elemental formula (*n *= 12) CS: prednisolone 2 mg/kg/d (maximum 60 mg/d) and sulphasalazine 25 mg/kg/d (*n *= 12)	12.9 (5.7–17.2)	Children with active CD	4	Activity was graded According to the Lloyd–Still activity index	3
Ruuska et al. [40]	Finland	EEN: whole-protein based on weight (*n *= 10) CS: prednisolone 1.5 mg/kg/d up to a maximum of 60 mg (*n *= 9)	8.5–18.6	Children with new onset or relapsing CD	8	PCDAI < 10	3
Borrelli et al. [41]	Italy	EEN: polymeric formula 120% to 130% of the recommended daily requirement (*n *= 19) CS: methylprednisolone 1.6 mg/kg/d (up to maximum 60 mg) for 4 wk, then tapered by 5–10 mg/kg, each week over 6 wk (*n *= 18)	4–17	Diagnosis of CD within 12 wk	10	PCDAI < 10	3
Retrospective analysis of records							
Canani et al. [42]	Italy	EEN: polymeric formula (*n *= 12); semi-elemental diet (*n *= 13); elemental diet (*n *= 12). 50–70 kcal/kg CS: methylprednisolone (1–2 mg/kg/d, maximal dose 40 mg/d) for 4 wk with subsequent gradual tapering over at least 4 wk (*n *= 10)	Polymeric: 11.5 (9–17) Semi-elemental: 11.8 (8–15) Elemental: 12.1 (7–16) CS: 12.4 (8–17)	Children with newly diagnosed CD	8	PCDAI < 10	8
Papadopoulou et al. [43]	Greece	EEN: elemental diet. The daily intake of the elemental diet ranged between 60 and 135 mL/kg/d, calculated to provide 140% of the recommended daily allowance of energy (*n *= 30) CS: prednisolone, 2 mg/kg/d up to a maximal dose of 60 mg/d, the dose was halved every 2–4 wk until a dose of 5–10 mg was achieved (*n *= 28)	EEN: 12.6 ± 3.1 CS: 12 ± 3.1	Children with diagnosed CD	8	Both the absence of clinical symptoms referable to Crohn’s disease (i.e., diarrhea, abdominal pain, fever) and achievement of a disease activity score > 80	8
Lambert et al. [44]	Australia	EEN: polymeric formula (*n *= 31). After completion of EEN, normal diet was reintroduced gradually and children were encouraged to continue supplementary volumes of enteral formula (500–1000 mL daily) CS: corticosteroids (*n *= 26).	EEN: 9.9 ± 4 CS: 9.97 ± 4.7	Children with newly diagnosed CD	6–8	PCDAI < 15	9
Soo et al. [45]	Canada	EEN: polymeric formula (*n *= 33) or semi-elemental formula (*n *= 3) for 6 wk and then partially over the next 2 wk depending on patient compliance CS: prednisone, 1 mg/kg/d to a maximum dose of 50 mg/d (*n *= 69)	EEN: 12.9 (7.4–16.2) CS: 11.2 (2.4–16.8)	Children with newly diagnosed CD	6–8	PCDAI < 10	9
Levine et al. [46]	Israel	EEN: polymeric formula (*n *= 43) CS: prednisone, 1–2 mg/kg up to 60 mg equivalent of prednisone (*n *= 114)	EEN: 12.3 ± 3.9 CS: 13.3 ± 3.1	Children with newly diagnosed CD	6–8	PCDAI < 10	8
Wang et al. [47]	China	EEN: polymer formula, according to the normal age of the required amount of 120–130% (*n *= 25) CS: oral methylprednisolone at an initial dose of 1.6 mg/kg/d (maximal dose ≤ 60 mg/d). After 4 wk of treatment, the dose was reduced (*n *= 23)	EEN: 9.3 ± 2.6 CS: 10.2 ± 3.1	Children with diagnosed CD	12	CDAI < 150 points or lower than the baseline value of at least 100 points and CRP normal	9
Hojsak et al. [48]	Croatia	EEN: polymeric formula, taken exclusively for 6–8 wk either through nasogastric tube or orally (*n *= 57) CS: corticosteroids (*n *= 17).	13.4 (1–17.9)	Children with newly diagnosed CD	6–8	PCDAI < 10	8
Grover et al. [49]	Australia	EEN: polymeric formula, Nutrison (1 kcal/mL, Nutricia, UK, 4 g protein, 3.9 g fat/100 mL) through nasogastric tube (NGT) or resource protein (1.25 kcal/mL, Nestle, 9.4 g protein, 3.5 g fat/100 mL) orally based on children preference and dietetic consultation (*n *= 43) CS: corticosteroids: 10 mg/d prednisolone (*n *= 46)	EEN: 13 (11.35–14) CS: 11.5 (9.5–13)	Children with newly diagnosed CD	6	PCDAI < 10	9
Luo et al. [50]	China	EEN: polymeric formula, the average caloric intake in EEN group was 117.9 ± 4.2 kcal (*n* = 10) CS: the average dosage for prednisone was 1.1 ± 0.4 mg/kg (*n* = 15). The average dosage for hydrocortisone was 8.7 ± 2.3 mg/kg (*n* = 3)	EEN: 11.1 (5–15) CS: 11.6 (1–16)	Children with active CD (PCDAI > 10)	8	PCDAI < 10	8
Hradsky et al. [51]	Czech Republic	EEN: polymeric formula (*n* = 29) CS: prednisone 1–2 mg/kg/d (up to 40 mg/d, exceptionally 60 mg/d) approximately 2 mont (*n* = 36)	EEN: 13.91 (12.3–15.02) CS: 14.85 (11.25–15.57)	Children with newly diagnosed CD	6–10	NG	7
Goncalves et al. [52] (abstract)	Portuguese	EEN: polymeric formula for 8 wk (1500–2000 mL/d) (*n* = 11) CS: the steroid dose was 1 mg/kg/d (*n* = 19)	There was no difference in age	Children with newly diagnosed CD	8	NG	7
Gavin et al. [53] (abstract)	UK	EEN: exclusive enteral nutrition (*n* = 43) CS: steroids (*n* = 19)	13 (2–16)	Children with newly diagnosed CD	NG	Clinical remission was determined using a physician global assessment and blood biochemistry	6
Scarpato et al. [54] (abstract)	Italy	EEN: receiving exclusive enteral nutrition for 8 wk, followed by a gradual introduction of foods during the subsequent 4 wk (*n* = 33). CS: treated with oral corticosteroids with tapering off by week 11 (*n* = 11).	EEN: 10.4 ± 3.5 CS: 11.7 ± 4.6	Children with newly diagnosed CD	8	NG	7
Prospective analysis of records							
Kierkus et al. [55]	Poland	EEN: each infused 1400–2200 mL. This provided approximately 50 kcal/kg/d (*n *= 20). CS: corticosteroids (*n *= 24)	EEN: 13.4 ± 5.18 CS: 13.8 ± 4.34	Children with moderate to severe CD (PCDAI > 30)	6	PCDAI < 10	9

*EEN* exclusive enteral nutrition group, *CS* corticosteroid group, *PCDAI* Pediatric Crohn’s Disease Activity Index, *CD* Crohn’s disease, *n* number, *NG* not given

### Quality assessment

The details of quality assessment based on the NOS are shown in Supplementary Table 1. The Newcastle–Ottawa scale revealed that study qualities varied from 7 to 9. The qualities of the included studies were good and fair. The quality of the four RCTs we included showed a moderate level. All articles displayed similar baselines and they were then grouped randomly. Patients included in the RCTs had complete follow-up information. The number of withdrawals and the reasons for withdrawal were described in the articles. However, only one RCT adopted the single-blind method, with three articles for three points and one article for five points.

### Effects of interventions

#### Induced remission rate of exclusive enteral nutrition vs. corticosteroids

**Fig. 2:**
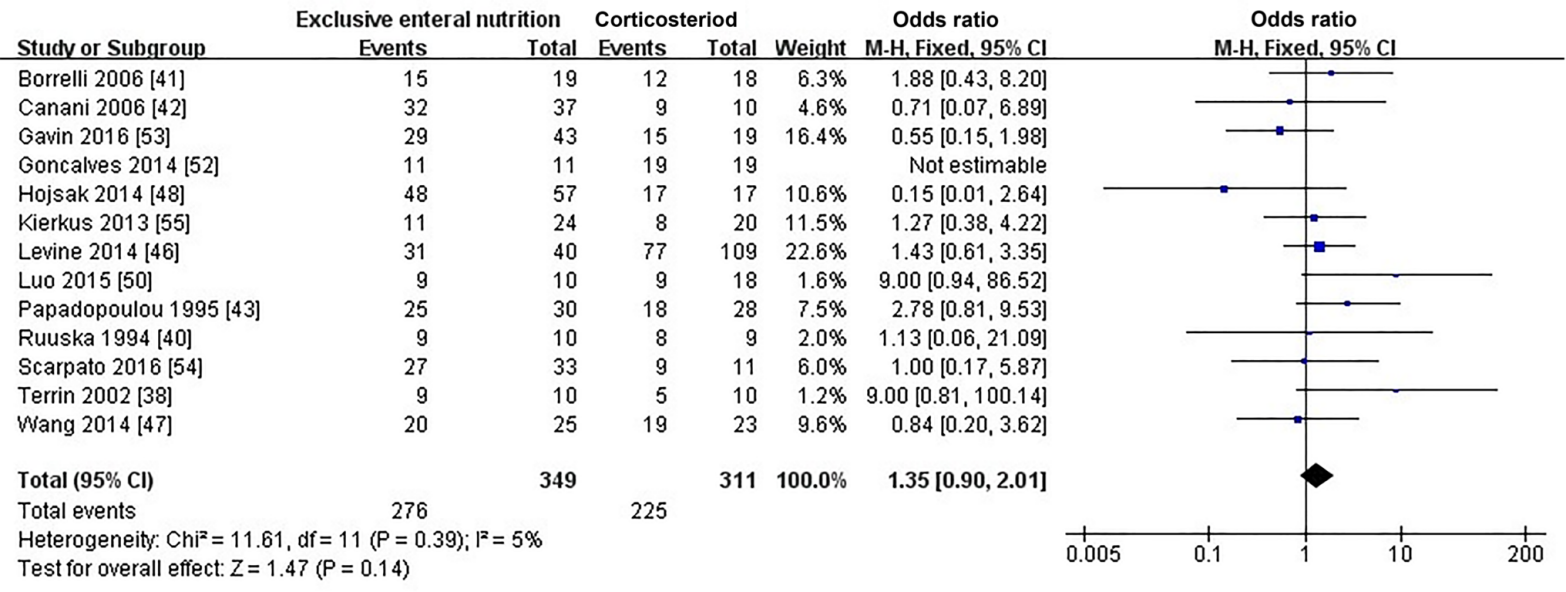
Forest plot for the comparison of induced remission rates between EEN versus corticosteroids for pediatric Crohn’s disease. No significant difference in induced remission rates was found between the EEN and corticosteroid groups (OR = 1.35; 95% CI 0.90, 2.10; *P *= 0.14). *EEN* exclusive enteral nutrition, *OR* odds ratio, *CI* confidence interval, *df* degree of freedom

Three RCTs [38, 40, 41] and ten observational studies [42, 43, 46–48, 50, 52–55] provided data on the induced remission rate after 6–8 weeks of treatment. Soo et al. [45] calculated the remission rate after 3 months treatment, so data from their paper were excluded. Overall, we analyzed 13 papers including 349 pediatric patients treated with exclusive elemental diet and 311 pediatric patients treated with corticosteroids. The heterogeneity test showed *I*^2^ = 5%, suggesting that there was a mild heterogeneity between the studies, so we performed the fixed-effect model in our study. We pooled all the results of these trials and found no evidence for a significant difference in the percentage of children achieving remission between those treated with EEN and those with corticosteroids (OR = 1.35; 95% CI 0.90, 2.10; *P *= 0.14; Fig. 2). Meta-analysis of only RCTs showed that EEN was more effective than corticosteroids (OR = 2.62; 95% CI 0.86, 7.94; *P *= 0.09, Supplementary Table 2).

#### Influence of exclusive enteral nutrition vs. corticosteroids on 1-year relapse rate

**Fig. 3:**
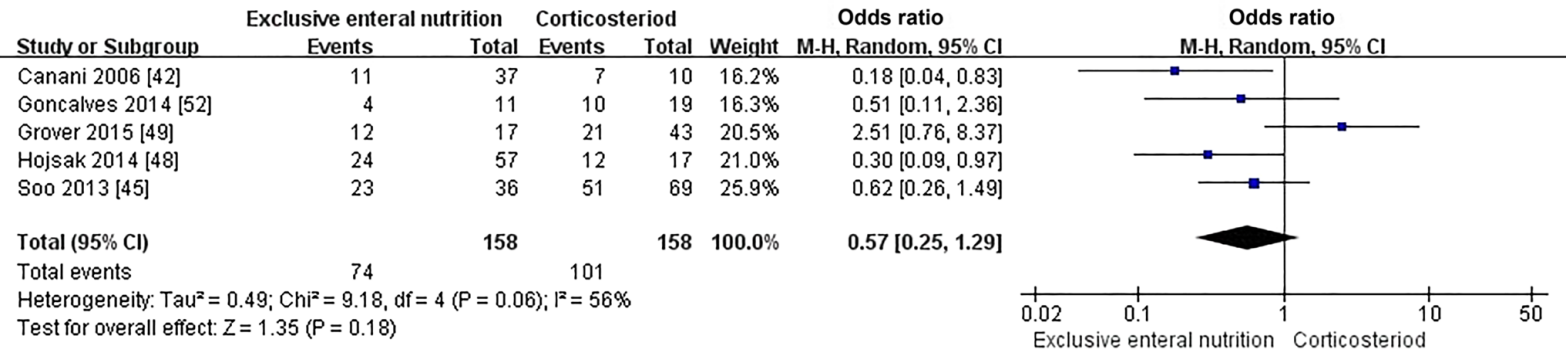
Forest plot for the comparison of 1-year relapse rates between EEN versus corticosteroids for pediatric Crohn’s disease. No significant difference in 1-year relapse rates was found between the EEN and corticosteroids groups (OR = 0.57; 95% CI 0.25, 1.29; *P* = 0.18). *EEN* exclusive enteral nutrition, *OR* odds ratio, *CI* confidence interval, *df* degree of freedom

Patients in five observational studies [42, 45, 48, 49, 52] were induced into remission with EEN or corticosteroids, and all of them used thiopurine and/or mesalamine as maintenance therapy. One study [44] that used maintenance enteral nutrition as maintenance therapy was excluded from the meta-analysis. There was only one RCT article, which was excluded as well. Furthermore, we included and analyzed five retrospective cohort studies (158 cases in the EEN group, 154 cases in the corticosteroids group). The meta-analysis of the five retrospective cohort studies also showed that no significant difference existed in the proportions of 1-year relapse rates between the EEN and corticosteroids groups (OR = 0.57; 95% CI 0.25, 1.29; *P *= 0.18, Fig. 3).

#### Mucosal healing

**Fig. 4:**
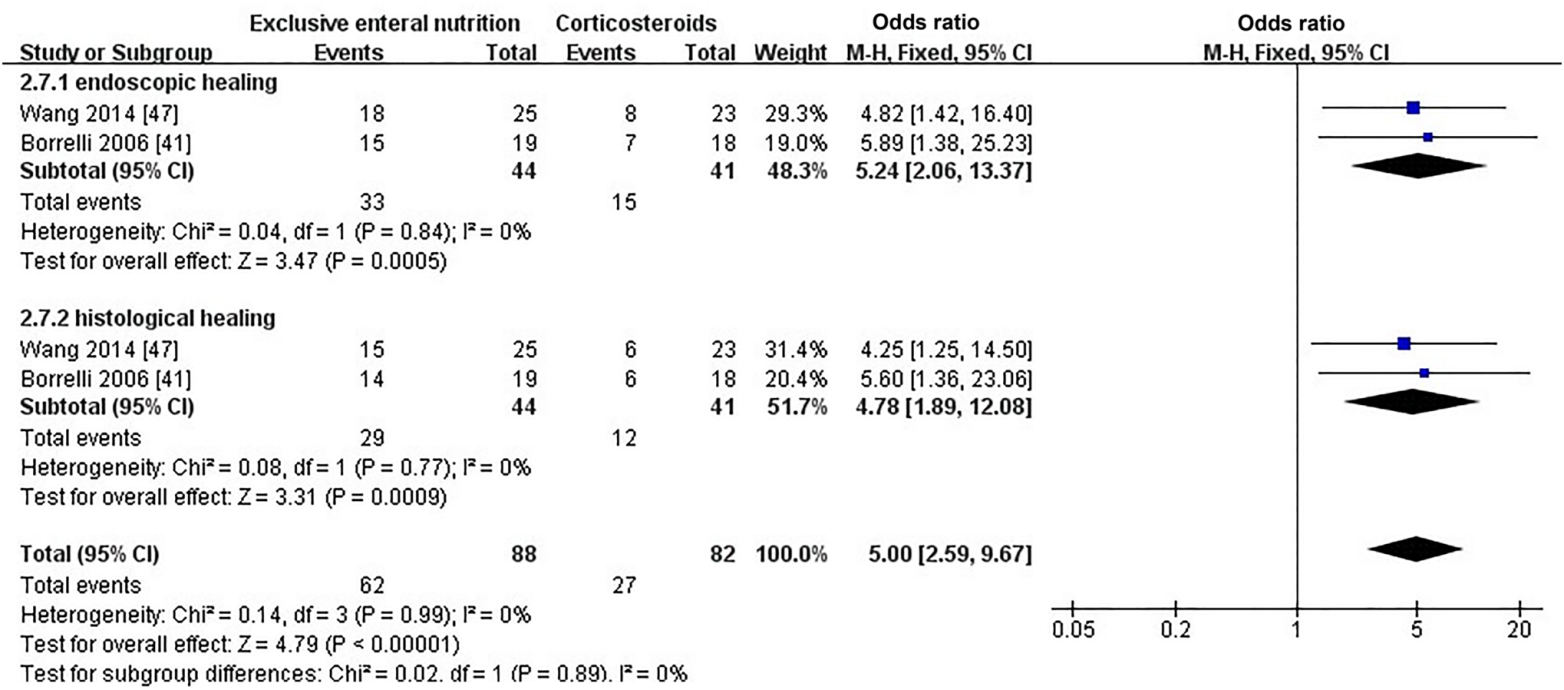
Forest plot for the comparison of mucosal healing between EEN versus corticosteroids for pediatric Crohn’s disease. A significant difference both in endoscopic and histological mucosal healing was found between the EEN and corticosteroid groups (OR = 5.24 0, 95% CI 2.06, 13.37; *P *= 0.0005 and OR = 4.78 0, 95% CI 1.89, 12.08; *P *= 0.0009, respectively). *EEN* exclusive enteral nutrition, *OR* odds ratio, *CI* confidence interval, *df* degree of freedom

In total, 3 of 18 articles [41, 42, 47] provided information on mucosal healing of patients at the end of induction. The method of Canani et al. [42] defining mucosal healing was different from other articles, so data from the research were excluded. Overall, patients who received EEN were more likely to achieve both endoscopic mucosal healing (OR = 5.24, 95% CI 2.06, 13.37; *P *= 0.0005, Fig. 4) and histological mucosal healing (OR = 4.78, 95% CI 1.89, 12.08; *P *= 0.0009, Fig. 4) than those who received corticosteroids.

#### Effects of exclusive enteral nutrition vs. corticosteroids on inflammation

**Fig. 5:**
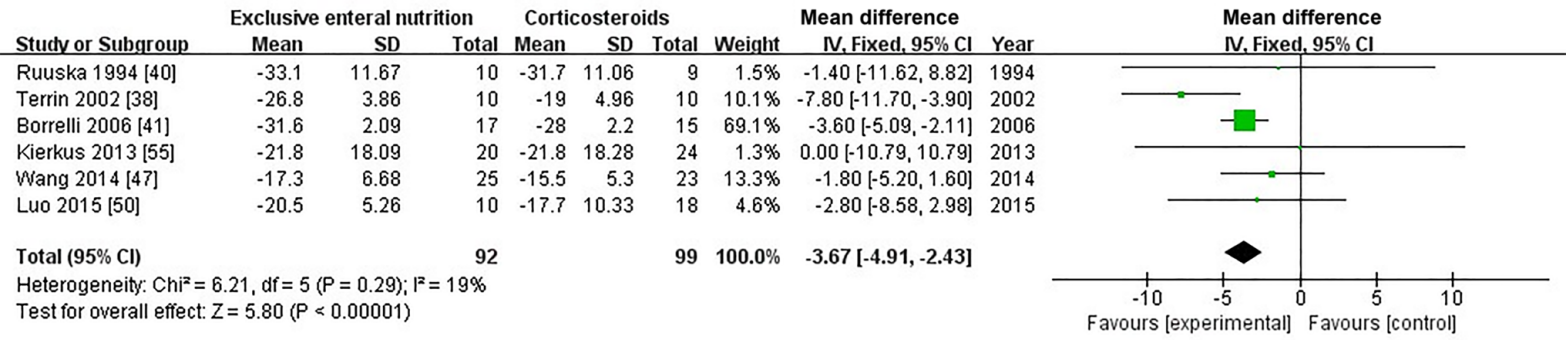
Forest plot for the comparison of PCDAI between EEN versus corticosteroids for pediatric Crohn’s disease. A significant difference in PCDAI was found between the EEN and corticosteroid groups with a standard mean difference of − 3.67 (95% CI − 4.91, − 2.43; *P *< 0.00001). *PCDAI* Pediatric Crohn’s Disease Activity Index, *EEN* exclusive enteral nutrition, *CI* confidence interval, *df* degree of freedom

The data were abstracted from studies that measured PCDAI, CRP and ESR at baseline and at the end of induction in patients receiving EEN and corticosteroids. Then, we used statistical algorithms to calculate the difference value’s mean and SD. There was a distinct decline of PCDAI in patients who received EEN compared with those who received corticosteroids (MD = − 3.67; 95% CI − 4.91, − 2.43; *P *< 0.0001, Fig. 5). However, we found no significant difference in terms of CRP and ESR between patients who received corticosteroids or EEN (MD = 1.07; 95% CI 0.18, 1.96; *P *= 0.02, on CRP, and MD = 0.60; 95% CI − 1.82, 3.03; *P *= 0.63, on ESR, Supplementary Figs. 1–2).

#### Effects of exclusive enteral nutrition vs. corticosteroids on growth

After induced therapy, patients who received EEN seemed to gain more weight than those who received corticosteroids (MD = 1.92; 95% CI = 0.02, 3.83; *P *= 0.05, Supplementary Fig. 3), but height showed no significant difference (MD = 0.24; 95% CI = − 1.98, 2.45; *P *= 0.83, Supplementary Fig. 4). We also found no significant difference at the 1-year end point in either height for age (MD = 0.45; 95% CI − 0.11, 1.02; *P *= 0.11, Supplementary Fig. 5) or BMI (MD = 0.10; 95% CI − 0.34, 0.54; *P *= 0.66, Supplementary Fig. 6).

## Discussion

Crohn’s disease is a chronic, progressive disease characterized by repeated relapses after remissions in most cases. The chronic intestinal inflammation that occurs in CD can result in intestinal complications such as strictures, fistulas, and abscesses during the whole period [56, 57]. Medical treatment of CD includes two major parts: induction and maintenance therapy. These phases of treatment involve achieving control of inflammation in a relatively short time and then sustaining that control to prevent patients from relapse. A number of investigators provided much evidence to illustrate the mechanism behind EEN. The primary components underlying the actions of EEN were as follows: inhibiting the expression of inflammatory factors, such as tumor necrosis factor-α, interleukin (IL)-6 and IL-1β [58–60], increasing the release of vascular endothelial growth factor and transforming growth factor-β (TGF-β) to improve intestinal endometrial repairment [61, 62], providing essential amino acids which can promote intestinal mucosal barrier formation [63, 64] and activating mucosal immunity, resulting in the maintenance of intestinal homeostasis [65].

Treatments for induced remission in CD patients include corticosteroids, EEN and biologic agents. Recently, people in many countries, such as the Japanese, Europeans, British, and North Americans, have highlighted the remission induction efficacy of EEN on pediatric CD [66–69]. There were two meta-analyses [15, 16] hinting that CS was superior to EEN in achieving control of inflammation in acute CD. These two meta-analyses were published in 1995 and 1996, and the retrieval times were between the 1980s and 1990s. The studies included in those two meta-analyses usually used EEN as remission induction therapy for fewer than 4 weeks, whereas the induction time of the studies we included mostly lasted for 6–8 weeks, with only one study lasting 4 weeks. In addition, those two meta-analyses included patients of all ages, and our meta-analyses focused on pediatric trials. Two other pediatric meta-analyses [17, 18] also determined that EEN and corticosteroids were equally effective, similar to our study’s conclusions, suggesting that the benefits of EEN may differ between children and adults.

No significant difference was found in 1-year relapse rates between EEN and corticosteroids. In our subgroup meta-analysis of the three articles [15–17], choosing thiopurine as single maintenance therapy had a similar result (EEN vs. CS: OR = 0.93, 95% CI 0.36, 2.43; *P* = 0.13). The use of thiopurine or mesalamine will impact the results, but it is also unethical to observe relapse rates without any maintenance therapy in child patients.

Recently, many researchers have regarded mucosal healing as a promising therapeutic target, and mucosal healing may substantially modify the course of CD. Mucosal remission was reported to be possibly associated with a sustained remission rate [26, 70]. Treatment with steroids was thought to have no positive impacts on mucosal healing [71]. In contrast, some studies reported approximately 19–75% of children treated with EEN achieved mucosal healing, but these results were unreliable since the definition of mucosal healing among the different studies varied [13, 72–74]. In our meta-analyses, there were two articles that used the CDEIS index to evaluate endoscopic mucosal healing, and the EEN group showed better outcomes than the CS group. A similar outcome was found for histological mucosal healing. A prospective longitudinal cohort study revealed that only complete mucosal healing (SES-CD = 0) post-EEN induction correlated with a lower sustained remission rate, and the sustained remission rate of near-complete mucosal healing patients (SES 1–3) was similar to that of patients with more active endoscopic disease [70]. Therefore, there is an urgent need to enlarge sample sizes, achieve cooperation between multiple centers and adopt uniform criteria to prospectively validate whether EEN is more effective in mucosal healing than CS and whether mucosal healing induced by EEN is conducive to sustained remission.

We also found that, after achieving induced remission, patients who received EEN treatment seemed to gain more weight than those who received corticosteroids. This result might be associated with nutritional support from EEN. In addition, we found that inflammation markers, such as CRP and ESR, showed no significant difference. However, patients who received EEN were 3.7 times more likely to have a decline of PCDAI score than those who received corticosteroids. The PCDAI is an index of severity of pediatric CD including physical examination, growth parameters and commonly performed laboratory tests [27]. The weight gain in the EEN group may be one factor contributing to PCDAI score decline. Despite the well-described adverse effects that corticosteroids can have on development, growth, and pubertal maturation, particularly if there has been a clinical course of frequent relapses resulting in inadequate nutrition and associated with repeated courses of steroid treatment [75], using corticosteroids as inducing treatment shows similar effects with EEN on the change of height for age and BMI at 1 year. This result may suggest that applying corticosteroids as short-term induction therapy does not have such an adverse impact on long-term growth and development in pediatric patients as expected. However, other growth and development indicators, such as bone age, bone density and gonad development, need to be further evaluated. Most importantly, doctors must still be very prudent in choosing appropriate and effective inducing therapy for CD pediatric patients to reduce repeated and long-term use of corticosteroids.

Our meta-analysis has several limitations. The observational studies were not designed randomly, and the influence of subjectivity among doctors or parents on the experimental results could not be ruled out; RCT experiments with multicenter, large-scale, strict double-blind and randomly allocated trials should be conducted in the future.

In conclusion, our study suggests the induction remission rate of EEN is similar to that of corticosteroids and that EEN and corticosteroid therapy had no significantly different effects on 1-year relapse rate. However, EEN is superior to corticosteroids in terms of positive effects on short-term mucosal healing and reduction of PCDAI. For these reasons, we recommend EEN as an effective and viable first-line treatment for induction of CD remission.

## Electronic supplementary material

Below is the link to the electronic supplementary material.

Supplementary material 1 (TIFF 110 kb)

Supplementary material 2 (TIFF 102 kb)

Supplementary material 3 (TIFF 84 kb)

Supplementary material 4 (TIFF 86 kb)

Supplementary material 5 (TIFF 96 kb)

Supplementary material 6 (TIFF 83 kb)

Supplementary material 7 (DOCX 24 kb)
