# Supplementary figures and images for: Exclusive enteral nutrition versus corticosteroids for treatment of pediatric Crohn’s disease: a meta-analysis
Source: World J Pediatr. 2019 Jan 21;15(1):26–36. doi: 10.1007/s12519-018-0204-0 (PMC6394648; doi:10.1007/s12519-018-0204-0)

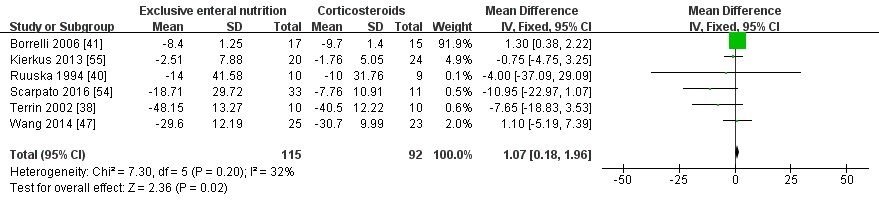

Supplement: Supplementary file 1 — Supplementary material 1 (TIFF 110 kb) [file 12519_2018_204_MOESM1_ESM.tif]

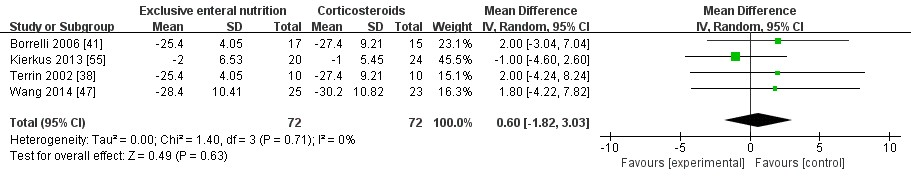

Supplement: Supplementary file 2 — Supplementary material 2 (TIFF 102 kb) [file 12519_2018_204_MOESM2_ESM.tif]

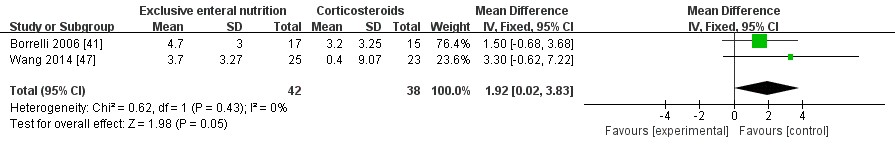

Supplement: Supplementary file 3 — Supplementary material 3 (TIFF 84 kb) [file 12519_2018_204_MOESM3_ESM.tif]

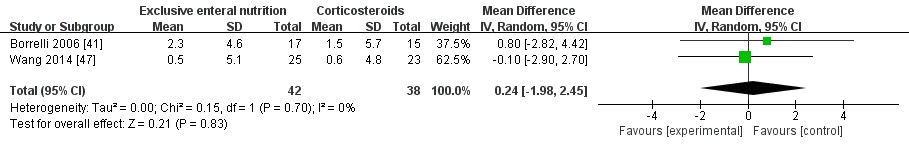

Supplement: Supplementary file 4 — Supplementary material 4 (TIFF 86 kb) [file 12519_2018_204_MOESM4_ESM.tif]

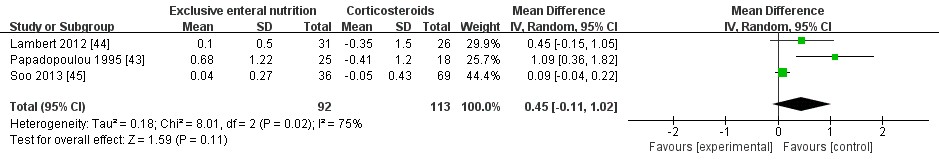

Supplement: Supplementary file 5 — Supplementary material 5 (TIFF 96 kb) [file 12519_2018_204_MOESM5_ESM.tif]

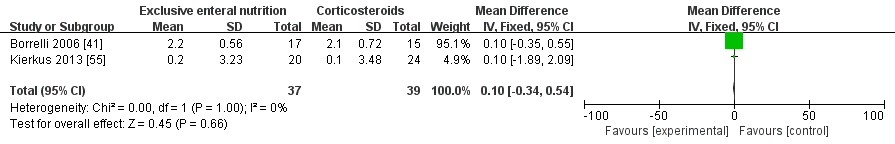

Supplement: Supplementary file 6 — Supplementary material 6 (TIFF 83 kb) [file 12519_2018_204_MOESM6_ESM.tif]
